# Autophagy and immunity – insights from human herpesviruses

**DOI:** 10.3389/fimmu.2012.00170

**Published:** 2012-07-04

**Authors:** Luke R. Williams, Graham S. Taylor

**Affiliations:** School of Cancer Sciences, University of Birmingham, Vincent Drive,Birmingham, UK

**Keywords:** antigen, CD4, EBNA1, EBV, macroautophagy, nucleophagy, presentation, processing

## Abstract

The herpesviruses are a family of double-stranded DNA viruses that infect a wide variety of organisms. Having co-evolved with their hosts over millennia, herpesviruses have developed a large repertoire of mechanisms to manipulate normal cellular processes for their own benefit. Consequently, studies on these viruses have made important contributions to our understanding of fundamental biological processes. Here we describe recent research on the human herpesviruses that has contributed to our understanding of, and interactions between, viruses, autophagy, and the immune system. The ability of autophagy to degrade proteins located within the nucleus, the site of herpesvirus latency and replication, is also considered.

## INTRODUCTION

In mammalian cells three different autophagy pathways have been shown to deliver cytoplasmic proteins or organelles into the lumen of lysosomes for degradation. The first pathway, microautophagy, is a process in which the lysosomal membrane invaginates, capturing portions of cytoplasm. Endosomal microautophagy has also recently been described (Sahu et al., 2011). The second pathway, chaperone-mediated autophagy (CMA), involves the transport of proteins containing a specific motif into the lysosome via the concerted actions of chaperone proteins and the lysosomal membrane protein LAMP2a (Dice, 2007). The third pathway, macroautophagy (hereafter referred to simply as autophagy) is the best described of the three and can be divided into six sequential steps: initiation, nucleation, elongation, closure, maturation, and degradation. Each step is a highly regulated process involving the coordinated action of numerous autophagy-related (ATG) and other proteins. Initiation of autophagy occurs upon activation of the ULK1 and UKL2 complexes following release of their inhibition by the mTOR complex. Nucleation defines the site for the autophagic membrane to begin to form in the cytoplasm and depends on the oligomerization of Beclin-1 that acts as a platform for recruitment of multiple other autophagy-related proteins. Elongation of the autophagic membrane around a portion of cytoplasm involves the recruitment of more proteins and lipidation of the microtubule-associated protein light chain 3 (LC3) by the E2-like enzyme ATG3. Closure of the membrane completes the formation of the double membrane vacuole, called an autophagosome, containing a portion of cytoplasm within its lumen. The autophagosome then undergoes maturation by fusing with lysosomes to form an autophagolysosome. This process is again mediated by Beclin-1 as well as LC3 on the autophagosome. Following maturation, the captured cytoplasmic contents are degraded by lysosomal proteases for reuse by the cell.

Autophagy typically occurs continuously at a low level but is increased in response to cellular stresses such as nutrient deprivation in order to provide a supply of nutrients to aid cell survival. In addition to this survival function, autophagy plays other important roles that are the focus of this review.

Viruses are obligate intracellular parasites and therefore totally dependent upon a host cell for replication. This dependence has led viruses to develop ways of harnessing normal cellular processes for their own purposes. A diverse range of viruses has now been shown to manipulate autophagy. Some viruses stimulate autophagy to aid their replication. Infection with poliovirus induces the formation of membranes resembling double-membrane autophagosomes that contribute to virus production (Suhy et al., 2000; Jackson et al., 2005). Rotavirus also stimulates the formation of autophagosome-like structures with the virus replicating in close proximity to these vesicles (Berkova et al., 2006). In contrast, autophagy poses a threat for other viruses and a range of viral countermeasures have been identified (Jordan and Randall, 2011). Autophagy inhibition has been well characterized for members of the herpesvirus family. In humans, eight herpesviruses have been identified that are divided into three sub-families (alpha-, beta-, and gammaherpesvirinae). These divisions were historically based on cellular tropism but are now based on viral genome organization (**Table 1**). Viruses across all sub-families are able to manipulate autophagy, employing a range of different mechanisms. These mechanisms and the effects on both viral replication and the host antiviral immune response are described below.

**Table 1:** Classification of the eight human herpesviruses.

Formal taxonomic name	Alternative common name	Viral sub-family
*Human herpesvirus* 1	Herpes simplex virus 1	*Alphaherpesvirinae*
*Human herpesvirus 2*	Herpes simplex virus 2	*Alphaherpesvirinae*
*Human herpesvirus* 3	Varicella-zoster virus	*Alphaherpesvirinae*
*Human herpesvirus* 4	Epstein–Barr virus	*Gammaherpesvirinae*
*Human herpesvirus* 5	Human cytomegalovirus	*Betaherpesvirinae*
*Human herpesvirus* 6	HHV-6 variant A or B	*Betaherpesvirinae*
*Human herpesvirus* 7	HHV-7	*Betaherpesvirinae*
*Human herpesvirus* 8	Kaposi’s sarcoma-associated herpesvirus	Gammaherpesvirinae

## HSV-1 INHIBITS AUTOPHAGY TO PREVENT VIRAL DEGRADATION

Autophagy has been shown to play important roles in many pathways of the innate immune system. These roles include modulating pathogen recognition receptors (Lee et al., 2007; Takeshita et al., 2008), controlling the production of key innate cytokines (Lee et al., 2007; Saitoh et al., 2008), and controlling phagocytosis (Sanjuan et al., 2007; Zang et al., 2012). Autophagy can directly contribute to innate immune control of viruses within infected cells, a role identified in the first study on Beclin-1 (Liang et al., 1998). Overexpression of Beclin-1 in neurons protected mice against fatal infection with the neurotropic alphavirus Sindbis (Note: Sindbis is a member of the *Togaviridae* family and not a herpesvirus; Liang et al., 1998). This protection resulted from the degradation of the Sindbis capsid protein by autophagy (Orvedahl et al., 2010). A range of intracellular bacteria and viruses are now known to be degraded by autophagy, a process termed xenophagy (Rich et al., 2003; Gutierrez et al., 2004; Nakagawa et al., 2004; Talloczy et al., 2006). The alphaherpesvirus herpes simplex virus type 1 (HSV-1) provided the first evidence of a pathogen inhibiting autophagy to escape xenophagy. Within virus-infected cells activation of cellular dsRNA-dependent protein kinase R (PKR) inhibits host and viral protein translation and viral replication. HSV-1 encodes a protein, ICP34.5, which inhibits this PKR-dependent antiviral activity (Chou et al., 1990). Talloczy et al. (2002) demonstrated that ICP34.5 also prevents PKR-dependent activation of autophagy in HSV-1-infected cells. Subsequent research revealed that ICP34.5 binds the essential autophagy gene Beclin-1 preventing the formation of autophagosomes (Orvedahl et al., 2007). Deletion of the Beclin-1 binding domain from ICP34.5 did not alter viral growth *in vitro* but could decrease viral growth and neurovirulence in a mouse model. Note that this decrease in neurovirulence could be due to a number of different factors. For example, survival of infected cells might be increased by the presence of a functional autophagy pathway. However, two observations support a contribution from xenophagy in reducing HSV-1 neurovirulence. First, HSV-1 virions can be observed in vesicles that morphologically resemble autophagosomes (Smith and de Harven, 1978; Talloczy et al., 2006). Second, biochemical studies show that inhibiting autophagy decreases the rate of degradation of HSV-1 proteins (Talloczy et al., 2006).

## HERPESVIRUSES DIFFER IN THEIR ABILITY TO INHIBIT AUTOPHAGY

Other herpesviruses have also been shown to possess autophagy inhibitory mechanisms. In cells infected with the betaherpesvirus human cytomegalovirus (HCMV) autophagy is initially activated before being inhibited 24 h post infection (Chaumorcel et al., 2008; McFarlane et al., 2011). HCMV-mediated autophagy inhibition requires *de novo* viral protein synthesis and is mediated by the viral protein TRS1 (Chaumorcel et al., 2012). Interestingly, TRS1 appears to be a functional homolog of the HSV protein ICP34.5, interacting with both PKR and Beclin-1. Like ICP34.5 it is the interaction between TRS1 and Beclin-1 that inhibits autophagy.

The human gammaherpesvirus Kaposi’s sarcoma-associated herpesvirus (KSHV) also inhibits autophagy by targeting Beclin-1 but uses a different mechanism to do so. The cellular Bcl-2 protein inhibits apoptosis but can also inhibit autophagy by binding Beclin-1(Pattingre et al., 2005). KSHV encodes a viral homolog of Bcl-2, called orf16, that can similarly bind Beclin-1 and inhibit autophagy (Pattingre et al., 2005). It is not currently known whether the two viral Bcl-2 homologs (BHRF1 and BALF1) encoded by the other human gammaherpesvirus Epstein–Barr virus (EBV) can similarly inhibit autophagy. However, it seems likely that one or both of these viral proteins will do so given that the viral Bcl-2 homolog encoded by another gammaherpesvirus, in this case murine MHV68, inhibits autophagy (Pattingre et al., 2005). Strong evidence for the importance of autophagy inhibition by these viral Bcl-2 homologs is provided by the MHV68 system. Mutating the Beclin binding domain of the MHV68 Bcl-2 homolog did not effect the establishment of viral latency but impaired the ability of the virus to maintain chronic infection in mice (E et al., 2009).

Interestingly, the protein interacting with carboxy terminal 1 (PICT-1), a putative human tumor suppressor protein of unknown function, has recently been shown to bind KSHV Bcl-2 (Kalt et al., 2011). Ectopic expression of PICT-1, which contains two nuclear localization sequences (NLS) and a nucleolar localization signal, caused KSHV Bcl-2 to relocalize from its normal mitochondrial localization into nucleoli thereby inhibiting its anti-apoptotic activity. The ability of PICT-1 to counteract KSHV Bcl-2-mediated autophagy inhibition was not examined in the paper. However, since Beclin-1 is almost entirely located in the cytoplasm and acts in this compartment (Liang et al., 2001), sequestration in the nucleus may well interfere with the ability of KSHV Bcl-2 to inhibit autophagy. If this proves to be the case then PICT-1 may represent the first example of a cellular countermeasure against viral inhibition of autophagy.

All of the herpesvirus autophagy inhibitory proteins described thus far act by interacting with Beclin-1. KSHV has developed an additional inhibitory mechanism, targeting a separate component of the cellular autophagy machinery. The viral FLICE like inhibitor protein (v-FLIP), a homolog of a cellular (c-FLIP), can inhibit both apoptosis and autophagy (Lee et al., 2009). v-FLIP binds Atg3 preventing it from processing LC3 thereby inhibiting autophagosome formation. Overexpression of either c-FLIP or v-FLIP inhibited cell death induced by rapamycin stimulation of autophagy (Lee et al., 2009).

In contrast to the herpesviruses described above varicella-zoster virus (VZV) is unable to inhibit autophagy. Indeed, autophagy is rapidly activated following VZV infection with many autophagosomes present in infected cells in vitro and in infected cells within skin vesicles (Takahashi et al., 2009; Carpenter et al., 2011). Unlike the other neurotropic alpha herpesviruses HSV-1 and HSV-2, VZV lacks an ICP34.5 ortholog; VZV also lacks a viral Bcl-2 homolog. The high level of autophagy present in VZV-infected cells may be the cause of the low titers of this virus obtained *in vitro*. It is interesting to note that an inability to inhibit autophagy has not proved detrimental to VZV; almost everyone is infected with the virus and in that regard it is a highly successful pathogen. Indeed, it has been postulated that VZV-induced autophagy may be beneficial to the virus, prolonging the survival of infected cells by reducing endoplasmic reticulum stress (Carpenter et al., 2011).

## AUTOPHAGY AND THE ADAPTIVE IMMUNE RESPONSE TO HERPESVIRUSES

Herpesviruses have evolved an arsenal of immune evasion mechanisms in order to establish life-long latent infection of immunocompetent hosts (Horst et al., 2011). As well as playing a key role in innate anti-viral immunity, autophagy is also important in adaptive immunity and autophagy manipulation by herpesviruses affects this arm of the immune response. In agreement with the earlier work suggesting xenophagy could reduce HSV-1 neurovirulence, strains of HSV-1 incapable of inhibiting autophagy (due to deletion of the Beclin-1 binding domain of ICP34.5) caused less disease following corneal infection of mice (Leib et al., 2009). However, no disease decrease was observed for *Rag1* knockout mice that lack B and T cells suggesting that the protective effect was mediated by adaptive, rather than innate, immunity. In accordance with this result mice infected with the mutant virus had higher virus-specific CD4+ T cell responses compared to animals infected with wild-type virus. While autophagy can reduce HSV-1-mediated disease the relative contributions made by the innate and adaptive immune responses are currently unclear and may differ depending upon the model system being used.

Viral inhibition of autophagy can potentially limit adaptive immunity by acting at two key stages. First, autophagy within virus-infected cells may be important for the generation of cross-primed CD8+ T cell responses by dendritic cells (DCs). Inhibiting autophagy in influenza-infected cells reduced their ability to stimulate influenza-specific CD8+ T cells in mice (Uhl et al., 2009). Similar results have also been observed using a tumor antigen model (Li et al., 2008). Second, autophagy provides a route for antigens to enter antigen-processing pathways within pathogen-infected cells, allowing them to be processed and presented to antigen-specific T cells. Since the first landmark observation that autophagy can generate an MHC II epitope from a self antigen (Brazil et al., 1997) several pathogen-encoded antigens have been shown to be processed this way. These antigens include the bacterial protein neomycin phosphotransferase (NeoR; Nimmerjahn et al., 2003), the mycobacterial antigen Ag85B (Jagannath et al., 2009), and EBV nuclear antigen 1 (EBNA1), the first example of a viral protein processed by autophagy for recognition by CD4+ T cells (Paludan et al., 2005). Autophagy can also generate MHC class I epitopes, the first example being arising from studies on HSV-1 infection of murine macrophages (English et al., 2009). As expected, autophagy was inhibited in the HSV-1-infected cells by ICP34.5. These infected cells also presented an MHC class I restricted viral epitope generated using the classical proteasome-dependent pathway. However, at later stages of infection higher levels of the epitope were generated and an additional processing pathway was involved that was sensitive to bafilomycin A and 3-methyladenine, inhibitors of the vacuolar processing pathway and autophagy respectively. In addition to normal double membrane autophagosomes, the HSV-infected cells also contained unusual four layered membrane structures originating from the nucleus that, based on the presence of LC3, appear to be autophagosomes. These unusual nuclear-derived autophagosomes were not observed in cells treated with rapamycin to stimulate autophagy nor cells infected with recombinant HSV-1 lacking ICP34.5. They may therefore represent a cellular response to a combination of HSV-1 infection and ICP34.5-mediated blockade of conventional autophagy. A second interesting observation from this work was that presentation of the viral MHC class I epitope by cells infected with HSV-1 lacking ICP34.5 was also sensitive to bafilomycin A and therefore involved the vacuolar pathway. As these cells lacked the four layered autophagosomes this result may indicate that classical double membrane autophagosomes also contribute to the generation of MHC class I epitopes.

## NUCLEAR SHELTER – ANOTHER STRATEGY TO EVADE AUTOPHAGY

Upon establishing latent infection, the herpesvirus genome persists as an episome within the nucleus. To ensure the viral genome is replicated and segregated into daughter cells the gammaherpesviruses EBV and KSHV each express a genome maintenance protein (EBNA1 and latency-associated nuclear antigen (LANA) respectively). Deletion of EBNA1 decreased EBV growth transformation 10,000-fold, clearly demonstrating the vital role of such proteins (Humme et al., 2003). Although loss of EBNA1 can be compensated by integration of the EBV genome into cellular DNA, this is a very rare event, again highlighting the importance of a correctly functioning genome maintenance protein for the gammaherpesviruses.

The dependence on the continuous presence of genome maintenance proteins to ensure life-long viral persistence represents a potential Achiles’ heel for these viruses. Logically, one would therefore expect genome maintenance proteins to be well protected from immunological surveillance. This is indeed the case. EBNA1 contains a large internal glycine/alanine repeat (GAr) domain that decreases EBNA1 processing and presentation by the HLA class I processing pathway via several complementary mechanisms (Levitskaya et al., 1997; Tellam et al., 2008; Apcher et al., 2009). The KSHV genome maintenance protein LANA also contains an internal repeat domain, in this case consisting of acidic residues, that similarly reduces this protein’s synthesis (Kwun et al., 2007) and degradation (Zaldumbide et al., 2007). In contrast to short-lived proteins, which are generally thought to be degraded by the proteasome, long-lived proteins are thought to be degraded by autophagy; measuring the degradation of long-lived proteins is a classical method for measuring the autophagy activity of cells (Klionsky et al., 2008). The genome maintenance proteins might therefore be potential substrates for autophagic degradation. This is indeed the case for EBNA1, which was the first viral protein identified as being degraded by autophagy (Paludan et al., 2005). Furthermore, two different EBNA1-specific CD4+ T cell clones were able to recognize EBV-transformed B lymphoblastoid cell lines (LCLs) in an autophagy dependant manner, demonstrating that such degradation could expose EBNA1 to the immune system. Remarkably, of all the EBV latent cycle proteins EBNA1 contains the greatest number of MHC class II epitopes and most individuals possess strong CD4+ T cell immunity against this protein (Munz et al., 2000; Leen et al., 2001; Hislop et al., 2007). Taken together, these observations appear paradoxical: how can EBV persist in MHC class II positive B cells despite strong CD4+ T cell responses specific for EBNA1, the protein critical for viral persistence?

In contrast to the observations described above, other research groups using CD4+ T cell clones specific for other EBNA1 epitopes found these cells were unable to recognize LCLs (Khanna et al., 1997; Mautner et al., 2004). In our laboratory, CD4+ T cell clones specific for two epitopes could recognize LCLs expressing natural levels of the EBNA1 protein while clones specific for a third epitope could not (Leung et al., 2010). The discordant results reported by different laboratories may therefore reflect real differences in the presentation of different epitopes from this protein. Although cell surface levels were low, control experiments confirmed that the two epitopes presented by LCLs were indeed endogenously accessing the MHC II pathway. Interestingly, only one epitope was autophagy-dependent, the other being processed by another intracellular route that is currently under investigation. Different epitopes from the same source protein being processed by different routes has been observed previously for influenza hemagglutinin (Sinnathamby and Eisenlohr, 2003). When EBNA1 was over-expressed as a nuclear protein, surface levels of the two naturally presented epitopes increased slightly, but the processing routes remained the same. However, expressing EBNA1 as a cytoplasmic protein, through mutation of its NLS, resulted in all three epitopes being processed by autophagy and presented at high levels by cells. This striking result was a direct result of EBNA1’s new cytoplasmic localization since addition of a heterologous NLS reversed the effect (Leung et al., 2010). While EBNA1 needs to be localized to the nucleus for its correct functioning in the virus life cycle, nuclear localization clearly brings an additional benefit, namely limiting EBNA1’s endogenous processing by macroautophagy and presentation to CD4+ T cells. Potentially “nuclear shelter” (Leung and Taylor, 2010) could also help reduce CD8+ T cell presentation of EBNA1. Reducing protein turnover may decrease the need for newly synthesized replacement polypeptides that are the dominant source of MHC class 1 peptides from EBNA1 (Lee et al., 2004; Tellam et al., 2004; Voo et al., 2004; Mackay et al., 2009).

Are other nuclear-localized proteins sheltered from macroautophagy like EBNA1? Studies using non-viral proteins suggest this may be the case. The huntingtin and ataxin-1 proteins each contain a large poly-glutamine repeat that can cause these proteins to aggregate in cells. Aggregates of huntingtin or ataxin-1 were degraded by autophagy in the cytoplasm but not in the nucleus (Iwata et al., 2005). A mutation in the NLS of a tumor antigen causing it to relocalize out of the nucleus enhanced its CD4+ T cell recognition (Wang et al., 1999). Although it is not known if this epitope was processed by autophagy, the observation is consistent with the EBNA1 data. In contrast to these examples, presentation of an autophagy-dependant epitope from the NeoR protein was not reduced by the addition of a heterologous NLS (Riedel et al., 2008). The reason for this different result is unclear, but one possibility is that it might reflect different rates of nuclear import. Previous work has shown that although the addition of a minimal NLS allows nuclear import, this occurs with slow kinetics unless additional flanking sequences are included (Rihs and Peters, 1989). The import rate of the NLS-containing NeoR protein has not been measured, however EBNA1 is rapidly imported following its translation suggesting that levels of cytoplasmic EBNA1 are likely to be very low (Kitamura et al., 2006). Finally, it is important to note that other endogenous MHC class II processing pathways exist. Some of these pathways involve the proteasome (Delmas et al., 2005; Tewari et al., 2005), others CMA (Zhou et al., 2005) while others have not been mechanistically defined. Antigen localization can affect processing by one of these alternative pathways. The CMV nuclear protein IE1 contains an MHC class II epitope that is not processed by autophagy but by a proteasome-dependent mechanism. Although relocalization of IE1 into the cytoplasm did not alter the degree of presentation of this epitope, the mechanism of presentation was altered (Delmas et al., 2005).

## HOW DO NUCLEAR-LOCALIZED ANTIGENS ENTER THE AUTOPHAGY PATHWAY?

The fact that some EBNA1 MHC class II epitopes are autophagy-dependent raises the interesting question of where in the cell does the nuclear-localized protein access the autophagic pathway? Can autophagy occur in the nucleus? The best-studied example of “nucleophagy” is Piecemeal Microautophagy of the Nucleus (PMN), a form of microautophagy that occurs in yeast (Roberts et al., 2003). During PMN, small pieces of the nucleus are pinched off into the yeast degradative vacuole; PMN is therefore a relatively gentle and selective process that can be tolerated by the cell. A much more dramatic example of nucleophagy has been observed in the filamentous fungus *Aspergillus oryzae*. Here, entire nuclei are degraded after being surrounded by large (1–2 μm diameter) autophagosomes (Shoji et al., 2010). While such drastic loss of genetic material would be lethal for any mononuclear cell, it can be tolerated within the multi-nucleated hyphae of this organism. Since autophagy is important for the growth of this fungus under starvation conditions, nucleophagy may be acting to recycle the constituents of redundant nuclei for use at growing hyphal tips.

Do mammalian cells undergo nucleophagy? The extent to which PMN takes place in other species is unknown, but homologs of the yeast genes essential for PMN, *Vac8*, and *Nvj1*, have not been identified in animal cells (Mijaljica et al., 2010). Granting non-specific macroautophagy access to the nucleus could result in irreversible loss of genetic material with catastrophic consequences for the cell. Indeed, to guard against such loss mammalian cells inhibit autophagy during mitosis, a time when chromosomes enter the cytoplasm following breakdown of the nuclear membrane (Eskelinen et al., 2002). However, nucleophagy has been observed in mammalian cells in select circumstances. In mouse macrophages HSV-1 infection triggers an unusual form of autophagy involving four-layer autophagosomes emerging from the nuclear envelope (English et al., 2009). It is not known whether these unusual autophagosomes can form in human cells infected with HSV-1, or if other herpesviruses can trigger their formation.

Other examples of nucleophagy occur in the context of genetic mutations. In Bloom’s syndrome, a rare cancer-predisposing disorder caused by mutation of the *BLM* gene, micronuclei bud from the nucleus into the cytoplasm (Yankiwski et al., 2000). The extent to which these cytoplasmic micronuclei are substrates of autophagy and the ability of normal cells to release micronuclei are currently not known. Finally, cells with fragile nuclei, caused by mutations in genes encoding nuclear lamina proteins, have been observed to contain perinuclear autophagosomes/autolysosomes-containing nuclear components (Park et al., 2009). Intriguingly, the authors report similar autophagosomes/autolysosomes in wild-type cells, although at a much lower frequency.

Autophagy is now known to exhibit greater selectivity for its substrates than once thought. Several autophagy adaptor proteins have now been shown to transport a range of substrates to autophagosomes for degradation. These substrates include aggregated or ubiquitinated proteins, damaged mitochondria, and intracellular bacteria (reviewed in Johansen and Lamark, 2011). Selective autophagy could be another route for nuclear proteins to enter cytoplasmic autophagosomes, one that could operate without causing genetic loss. One of the autophagy adaptor proteins, p62 (also called SQSTM1), has recently been shown to deliver capsids of Sindbis virus to autophagosomes (Orvedahl et al., 2010). With respect to the selective autophagy of nuclear proteins, p62 is of particular interest since it contains both nuclear localization and nuclear export sequences and consequently shuttles rapidly between the nucleus and cytoplasm (Pankiv et al., 2010). However, it is not yet known whether p62 is involved in the selective degradation of nuclear proteins by autophagy.

## ACTIVATION OF AUTOPHAGY BY HERPESVIRUSES

Thus far we have considered autophagy as a threat to herpesviruses. However, the relationship between herpesviruses and autophagy is more complex and several examples exist of herpesviruses activating autophagy at different stages in their lifecycle. Autophagy activation can occur during lytic infection. As described earlier autophagy is rapidly activated in VZV-infected cells because the virus apparently lacks mechanisms to inhibit this pathway. However, autophagy activation has also been reported for viruses that do possess such mechanisms, suggesting that autophagy may be beneficial for viruses at particular stages of their replicative cycle. Although HSV-1 encodes the autophagy inhibitor ICP34.5 this virus has been reported to rapidly activate autophagy in human fetal foreskin fibroblasts (McFarlane et al., 2011). Autophagy induction by HSV-1 has also been reported in murine macrophages, although in this case classical macroautophagy still appeared to be inhibited by virus-encoded ICP34.5 and a morphologically distinct type of macroautophagy was observed (English et al., 2009). CMV infection also rapidly activates autophagy that is subsequently inhibited by the CMV-encoded protein TRS1 (McFarlane et al., 2011; Chaumorcel et al., 2012). KSHV also appears to activate autophagy during lytic reactivation. The KSHV protein RTA is able to activate autophagy, inhibiting this process reduced RTA-mediated lytic gene expression and KSHV replication suggesting autophagy plays a key role in the replication of KSHV during lytic cycle (Wen et al., 2010). Autophagy activation can also occur during herpesvirus latency. The EBV-encoded latent membrane protein 1 (LMP1) induces autophagy in EBV transformed B cells (Lee and Sugden, 2008). This induction does not result from LMP1’s well-documented activation of cellular signaling pathways but from the presence of three of the molecule’s transmembrane spanning domains. Since LMP1 is also degraded by autophagy these domains, which had previously not been assigned a functional role, may provide LMP1 with a feedback mechanism that regulates cellular levels of the protein.

## CONCLUSION

During their evolution, the herpesviruses have developed a range of mechanisms to interact with the host autophagy machinery. Under some circumstances these viruses need to evade host immunity and this is achieved in several ways. One way is to target key cellular proteins required for autophagy. In this regard, several viruses have independently evolved mechanisms that target Beclin-1, reflecting the key role this protein plays in autophagy. The targeting of another autophagy protein, ATG3, by KSHV raises the possibility that a range of inhibitory mechanisms exist, waiting to be discovered. Another way to evade immunity may be through localization of genome maintenance proteins into the nucleus, thereby minimizing their access to the autophagy machinery. In this review, we have focused on macroautophagy, which is the best characterized of the three currently defined autophagy pathways. The other pathways are beginning to be elucidated and one of them, CMA, has been shown to generate an MHC class II restricted epitope from a non-viral antigen (Zhou et al., 2005). However, the contribution of these other pathways to viral immunity, and whether they too are inhibited by herpesviruses, is currently unknown. Finally, a complex balance exists between herpesviruses and autophagy within the host cell and under some circumstances herpesviruses activate autophagy, presumably for their benefit. Two recent observations – PICT-1-mediated relocalization of KSHV Bcl-2 away from its site of action, and nuclear-derived autophagosomes in mouse macrophages infected with HSV-1 – raise the possibility that cells possess mechanisms to counteract viral manipulation of autophagy. Understanding the balance between autophagy inhibition and activation within virally infected cells, and the consequences for anti-viral immunity, presents a challenge and an opportunity for future research.

## Conflict of Interest Statement

The authors declare that the research was conducted in the absence of any commercial or financial relationships that could be construed as a potential conflict of interest.
